# Construction and evaluation of a machine learning-based predictive model for enteral nutrition feeding intolerance risk in ICU patients

**DOI:** 10.3389/fnut.2025.1600319

**Published:** 2025-07-09

**Authors:** Gaimei Wang, Cendi Lu, Owusu Mensah Solomon, Yujia Gu, Yijing Ling, Fanchi Xu, Yumin Tao, Yehong Wei

**Affiliations:** ^1^Department of Neurosurgery Unit, The Second Affiliated Hospital of Zhejiang Chinese Medical University, Hangzhou, China; ^2^International Education College, Zhejiang Chinese Medical University, Hangzhou, China; ^3^College of Nursing, Zhejiang Chinese Medical University, Hangzhou, China; ^4^Ningbo Municipal Center for Disease Control and Prevention, Ningbo, China; ^5^Intensive Care Unit, The Second Affiliated Hospital of Zhejiang Chinese Medical University, Zhejiang, China

**Keywords:** ICU patients, enteral nutrition, feeding intolerance, machine learning, prediction model

## Abstract

**Objective:**

We aim to investigate the factors influencing enteral nutrition feeding intolerance (ENFI) in critically ill patients and develop a risk prediction model for ENFI in intensive care unit (ICU) patients, utilizing three machine learning algorithms. This model will serve as an assessment tool for preventing and managing ENFI in ICU patients.

**Methods:**

A total of 487 ICU patients from a tertiary hospital in Zhejiang Province between January 2021 and December 2023 were selected as the study subjects. The patients were randomly divided into a training set and a test set in an 8:2 ratio. Three machine learning algorithms—logistic regression (LR), support vector machine (SVM), and random forest (RF)—were used to construct the risk prediction model for ENFI in ICU patients. The predictive performance of the three models was compared using metrics such as AUC (area under the ROC curve), accuracy, precision, recall, and F1 score.

**Results:**

The logistic regression model achieved an AUC of 0.9308, with an accuracy of 94.3%, precision of 95.4%, recall of 88.6%, and an F1-score of 0.9185 in correctly identifying ENFI risk in ICU patients. The random forest model attained an AUC of 0.9511, with an accuracy of 96.1%, precision of 97.7%, recall of 91.4%, and an F1-score of 0.9446. The support vector machine (SVM) model yielded an AUC of 0.9241, with an accuracy of 94.1%, precision of 96.8%, recall of 86.4%, and an F1-score of 0.9132.

**Conclusion:**

The random forest model performed the best in this study, demonstrating superior predictive performance.

## 1 Introduction

Early enteral nutrition (EN) support is a crucial component of comprehensive treatment for ICU patients. It provides essential nutrients and helps maintain the integrity of the intestinal mucosal barrier, reduces hypercatabolism, and prevents secondary infections (1). However, the occurrence of enteral nutrition feeding intolerance (ENFI) severely impacts the delivery of enteral nutrition (2). ENFI (3) is a term for gastrointestinal problems like abdominal distension, diarrhea, and constipation during EN. These problems cause the patient to stop or suspend enteral nutrition, which keeps them from meeting their target caloric intake within 72 h. This increases the incidence of malnutrition and prolongs the duration of mechanical ventilation and ICU stay, thereby increasing the medical burden (4).

Recently, with in-depth research on EN, it has been found that early identification and precise prevention of ENFI can optimize EN management and improve clinical outcomes (5). Most existing ENFI risk prediction models are based on traditional logistic regression analysis, which assumes a linear relationship between independent and dependent variables (6, 7). However, in real-world scenarios, many independent variables have nonlinear or locally approximate linear effects on individual risk functions, which can reduce the model’s effectiveness to some extent (8). With the advancement of computer science, machine learning algorithms are increasingly being applied in the medical field (9). Machine learning-based prediction models can fully exploit data characteristics and explore complex relationships and patterns within the data, providing strong support for disease prevention, diagnosis, and treatment (10). In this study, three ML algorithms widely used in the medical field are selected, and this class of algorithms demonstrates strong analytical processing capabilities in handling medical data. The LR algorithm is simple in principle, effective at dealing with linear classification problems (e.g., disease diagnosis), with small sample size requirements, but easy to overfit (11). The algorithmic principle of the RF algorithm is more complex, and it is susceptible to overfitting problems due to the influence of training data noise, but its performance is stable in solving the classification problem, and the results have a certain degree of interpretability (12). SVM has better generalization ability and robustness and can achieve better classification results with a limited training set, but the computational cost is high and the memory demand is large (13).

Considering the limitations of the aforementioned models, this study will use three machine learning algorithms to construct a risk prediction model for ENFI in ICU patients. It aims to help clinicians identify high-risk patients, enabling timely preventive interventions to reduce EFI incidence.

## 2 Methods

### 2.1 Study population

**Figure 1:**
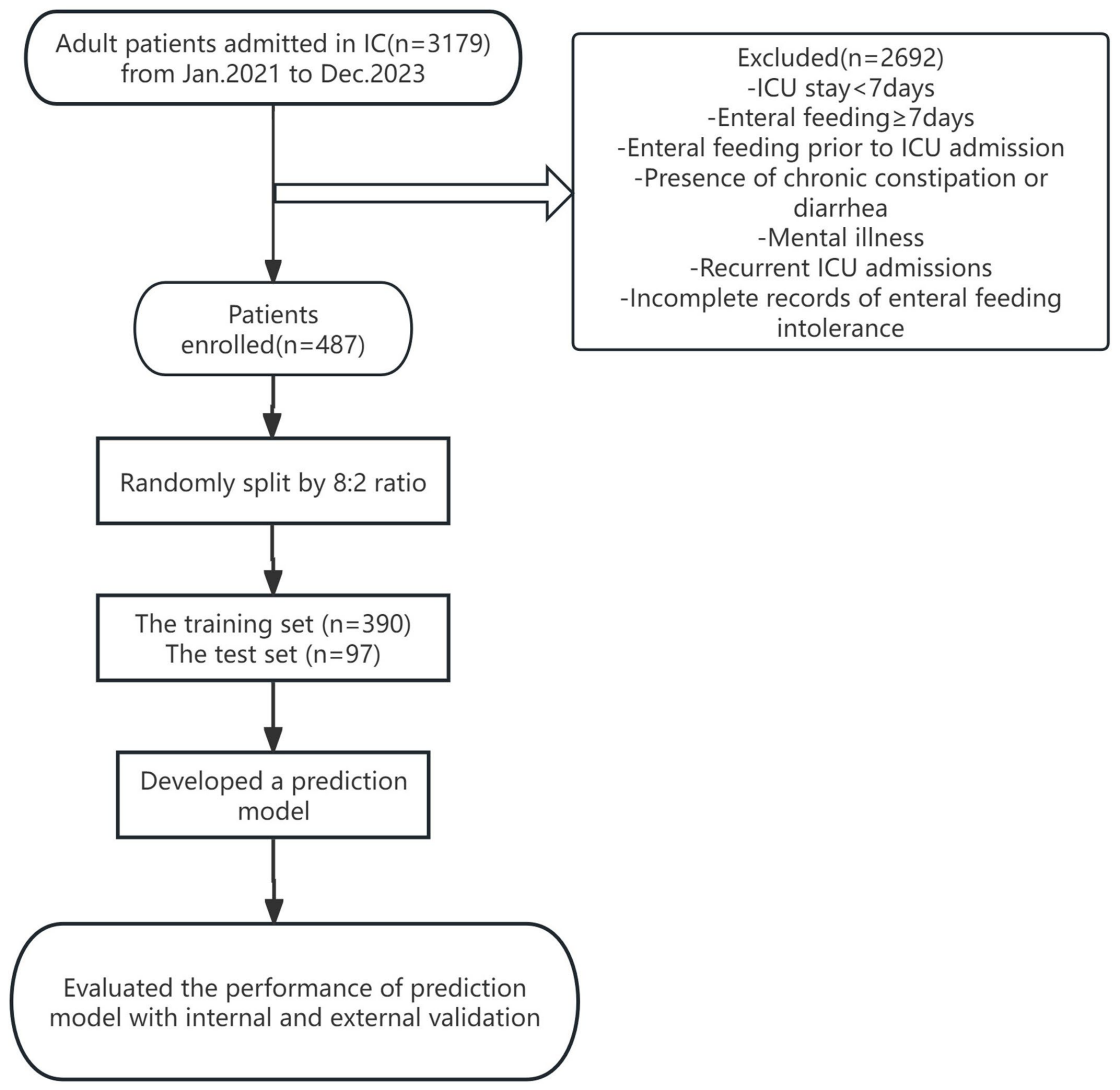
Flowchart of ICU patient enrollment and predictive model development.

A total of 3,179 patients admitted to the ICU of a tertiary hospital in Zhejiang Province from January 2022 to December 2023 were selected as the study subjects by convenience sampling, and 487 patients were finally included after screening. The specific process is detailed in Figure 1. The study was approved in written form by the Ethics Committee of the Second Affiliated Hospital of Zhejiang Chinese Medicine University under the approval number No. 050–01 of 2024, The Second Affiliated Hospital of Zhejiang Chinese Medical University. We de-identified the records for this study and waived informed consent, as outlined in the Declarations. Inclusion criteria: age ≥ 18 years; enteral nutrition initiated within 48 h of ICU admission. Exclusion criteria: history of gastrointestinal diseases or gastrointestinal surgery; enteral nutrition initiated before ICU admission; intra-abdominal pressure (IAP) ≥ grade III at ICU admission; inability to place a urinary catheter due to bladder or urethral conditions. This is a predictive modeling study using retrospective data, designed and reported following the TRIPOD+AI guideline for developing and validating multivariable prediction models.

### 2.2 Data collection

#### 2.2.1 Questionnaire on influencing factors of ENFI in ICU patients

**Figure 2:**
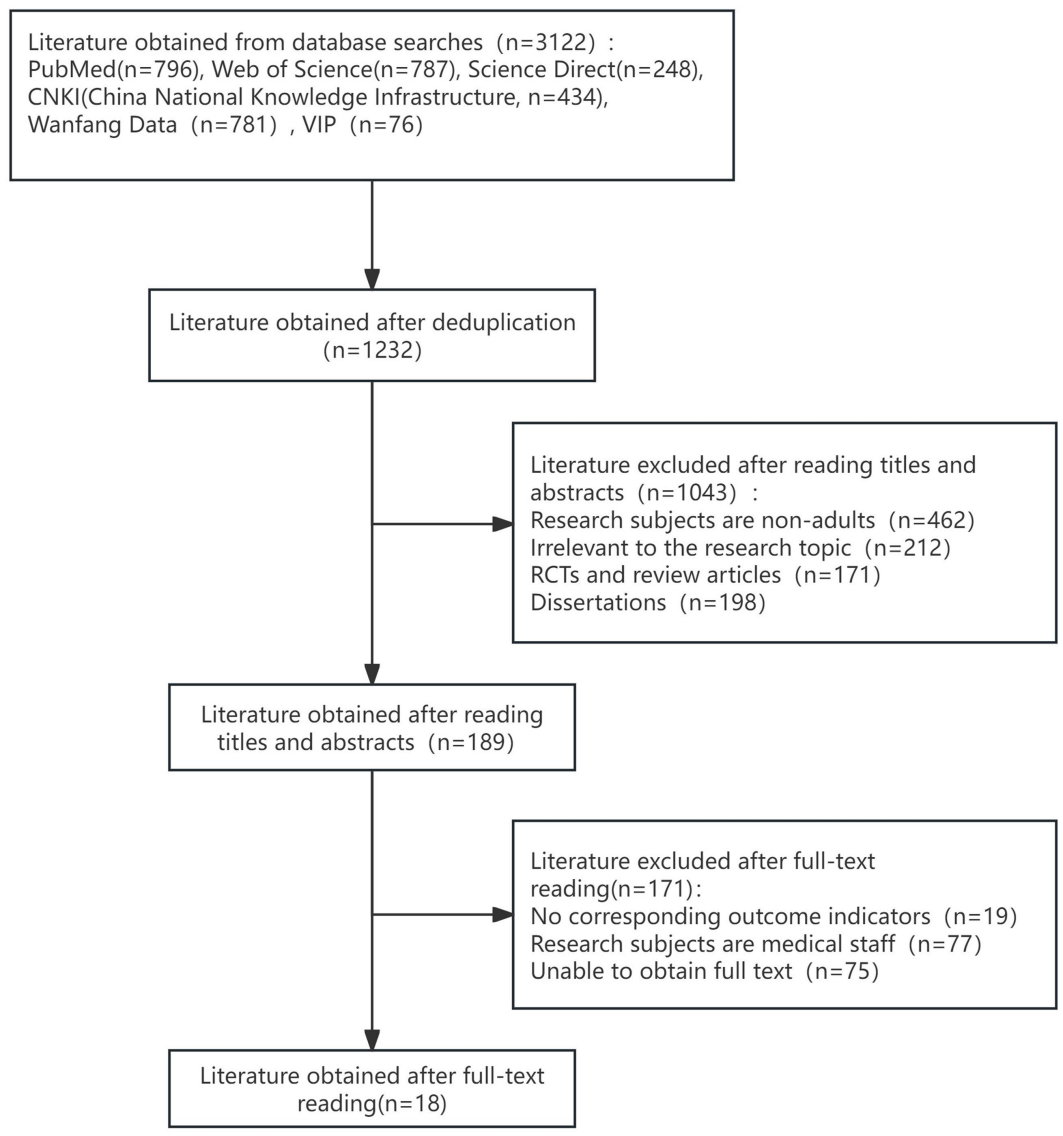
PRISMA flowchart of literature selection for ENFI risk factors.

To identify risk factors for ENFI in intensive care unit patients, this study conducted a systematic search of the databases PubMed, Web of Science, and China Knowledge Network (CNKI) from the inception of the databases to January 30, 2024, using the Medical Subject Headings (MeSH) and free-text terms. Two members of the research team independently screened the literature based on the Joanna Briggs Institute (JBI) checklist and the Johns Hopkins University Evidence Assessment Criteria, with disagreements adjudicated by a third party. The literature-specific screening process is shown in the PRISMA flowchart (Figure 2). Eighteen candidate risk factors were extracted from 18 selected high-quality articles. These risk factors were refined through two rounds of the expert meeting method, resulting in the identification of 26 potential risk factors for enteral nutrition intolerance. These factors included clinical baseline data, biochemical markers, and intervention-related variables, and the specific entries are detailed in Tables 1, 2. This multistage approach ensured the methodological rigor and clinical relevance of the study. To ensure the accuracy and stability of the model, the sample size was calculated based on the requirement that it should be at least 5–10 times the number of independent variables. Considering 26 influencing factors, an ENFI incidence rate of 38%, and a 10% sample attrition rate, at least 376 samples were required. In practice, this study included 487 samples, meeting the sample size requirement. We divided the samples into a training set and a test set in an 8:2 ratio.

**Table 1:** Comparison of factors influencing ENFI in ICU patients (non-normal distribution, *N* = 487).

Item	ENFI (*N* = 175)	Non-ENFI (*N* = 312)	Z(*P*)
Age (years)	84(75, 89)	77.5(67, 85.8)	−4.823(<0.001)
BMI	21.5(20.4, 22.5)	21.6(20.4, 22.7)	−0.828(0.408)
APACHE II score	34(25, 43)	20(14, 23)	−13.386(<0.001)
NRS-2002	5(4, 6)	4(4, 5)	−7.753(<0.001)
IAP (mmHg)	12(9, 12)	8(6, 9)	−15.44(<0.001)
CPOT pain score	3(2, 4)	2(1, 3)	−7.854(<0.001)
Albumin (g/L)	26.5(23.1, 30.2)	30.75(27.5, 33.4)	−7.849(<0.001)
Bloo*d* glucose (mmol/L)	8.9(6.7, 11.3)	6.3(5.2,8.3)	−7.275(<0.001)

Data are expressed as median P50(P25, P75); *p* < 0.05 is considered a statistically significant difference.

**Table 2:** Comparison of factors influencing ENFI in ICU patients (categorical variables, *N* = 487).

Item	Form	ENFI (*N* = 175)	Non-ENFI (*N* = 312)	χ^2^(P)
Sex	Male	60(34.3)	210(67.3)	0.128(0.72)
	Female	12(6.9)	102(32.7)	
Primary diagnosis	Circulatory System	103(58.9)	14(4.5)	16.696(0.019)
	Respiratory system	1(0.6)	140(44.9)	
	Digestive system	1(0.6)	6(1.9)	
	Endocrine system	50(28.6)	8(2.6)	
	Nervous system	1(0.6)	135(43.3)	
	Urinary System	7(4)	2(0.6)	
	Blood System	92(52.6)	7(2.2)	
Hemodynamics	Stable	83(47.4)	271(86.9)	69.449(<0.001)
	Unstable	90(51.4)	41(13.1)	
Sedation	Yes	85(48.6)	73(23.4)	39.561(<0.001)
	No	34(19.4)	239(76.6)	
Early activity	Yes	141(80.6)	82(26.3)	2.902(0.088)
	No	149(85.1)	230(73.7)	
Use of mechanical ventilation	Yes	26(14.9)	208(66.7)	20.145(<0.001)
	No	38(21.7)	104(33.3)	
Use of CRRT	Yes	137(78.3)	39(12.5)	7.151(0.007)
	No	90(51.4)	273(87.5)	
Use of sedation	Yes	85(48.6)	75(24)	37.546(<0.001)
	No	68(38.9)	237(76)	
Use of analgesics	Yes	107(61.1)	29(9.3)	61.427(<0.001)
	No	61(34.9)	283(90.7)	
Use of vasoactive drugs	Yes	114(65.1)	56(17.9)	17.561(<0.001)
	No	131(74.9)	256(82.1)	
Use of antibiotics	Yes	44(25.1)	220(70.5)	1.051(0.305)
	No	64(36.6)	92(29.5)	
Use of Potassium	Yes	111(63.4)	39(12.5)	38.954(<0.001)
	No	67(38.3)	273(87.5)	
Use of probiotics	Yes	108(61.7)	167(53.5)	10.432(0.001)
	No	54(30.9)	145(46.5)	
Use of gastrointestinalstimulants	Yes	121(69.1)	145(46.5)	11.316(0.001)
	No	71(40.6)	167(53.5)	
Use of laxatives	Yes	104(59.4)	53(17)	32.858(<0.001)
	No	68(38.9)	259(83)	
Early enema	Yes	107(61.1)	34(10.9)	52.933(<0.001)
	No	23(13.1)	278(89.1)	
Types of nutritional solution	Beplix	39(22.3)	32(10.3)	12.899(0.115)
	Nutriforce	3(1.7)	105(33.7)	
	Kangquan	29(16.6)	6(1.9)	
	Risen	47(26.9)	51(16.3)	
	Ridai	1(0.6)	68(21.8)	
	Risperdal	6(3.4)	2(0.6)	
	Rexall	27(15.4)	2(0.6)	
	Renesas	111(63.4)	46(14.7)	
Route of enteral nutrition	Transgastric tube route	61(34.9)	249(79.8)	27.994(<0.001)
	Transintestinal Tube Route	3(1.7)	56(17.9)	
	Transgastric fistula route		7(2.2)	

Data are expressed as number of cases (%); *P < 0.05 is considered a statistically significant difference.

#### 2.2.2 Assessment of ENFI

According to the diagnostic criteria for enteral nutrition feeding intolerance (ENFI) established by the Abdominal Problems Working Group of the European Society of Intensive Care Medicine (15), combined with clinical practice, the diagnostic criteria for ENFI in ICU patients were defined as follows: (1) Failure to achieve the target caloric intake of at least 20 kcal/(kg·d) within 72 h after initiating enteral nutrition; (2) Suspension or discontinuation of enteral nutrition due to gastrointestinal symptoms including abdominal distension (IAP ≥ 12 mmHg), vomiting (expulsion of gastric contents through the mouth occurring once or more), or diarrhea (≥3 episodes of loose watery stools per 24-h period, with each stool volume >200 g); or (3) Gastric residual volume (GRV) monitoring every 6 h, with either a single GRV measurement ≥200 mL or cumulative GRV exceeding 500 mL within 24 h. In this study, GRV was established as a secondary observational indicator, which is not used alone to assess enteral nutrition intolerance, but needs to be used in conjunction with other outcome indicators. This indicator was retained based on institutional research protocols, but according to the American Society for Parenteral and Enteral Nutrition (ASPEN) recommendations, GRV needs to be interpreted with caution and weighted strictly in the assessment system to ensure the scientific validity of the study conclusions. The assessment, based on the medical records from the first 7 days of enteral feeding, was uniformly applied to both the training and validation sets. It was conducted independently by a nutrition nurse specialist, a critical care specialist, and the researcher, with the diagnosis of ENFI requiring agreement from at least two of the evaluators.

### 2.3 Data processing

All predictor variables (e.g., APACHE II scores, IAP) were collected before or at the time of enteral nutrition initiation, while ENFI outcomes were assessed within the subsequent 7 days. This temporal sequence ensures the model’s applicability for early risk prediction. *Data with missing rates exceeding 50% were excluded from the analysis (14), while data with missing rates below 50% were imputed using the random forest method via the “missForest” package in R (Handling missing data in a rheumatoid arthritis registry using a random forest approach—PubMed*, no date*)*.

### 2.4 Statistical methods

SPSS 25.0 statistical software was used for data analysis. For continuous variables with a normal distribution, descriptive statistics were shown as mean ± standard deviation (mean ± SD), and t-tests were used to see if there were any differences between the groups. For continuous variables without a normal distribution, we used the median and interquartile range to present descriptive statistics. The Mann–Whitney U test was used to compare differences between groups that were not parametric. For categorical variables, frequencies and percentages were used for descriptive statistics, and chi-square tests were used to analyze differences between groups. Binary logistic regression was used to further screen influencing factors, with variables with *p* < 0.05 included in subsequent analyses. Python 3.9 was used to build and evaluate the prediction models, with the dataset divided into an 80% training set and a 20% test set. The predictive performance of the three models (LR, SVM, and RF) was compared using the training and test sets.

## 3 Results

### 3.1 Incidence of ENFI in ICU patients

The incidence of ENFI in the ICU patients in this study was 35.9%, with 175 out of 487 patients experiencing it. Among the 487 patients, 325 were male (66.7%) and 162 were female (33.3%). The average age was 76.76 ± 13.71 years, with a minimum age of 22 and a maximum age of 101. The average BMI was 21.78 ± 1.82, with a minimum of 16.4 and a maximum of 29.3. The top three primary diagnoses were respiratory diseases (243 cases, 49.9%), neurological diseases (185 cases, 37.9%), and circulatory system diseases (26 cases, 5.3%).

### 3.2 Univariate analysis of influencing factors of ENFI in ICU patients

Univariate analysis was conducted on the influencing factors of ENFI in ICU patients. Continuous variables were tested for normal distribution using the Kolmogorov–Smirnov (K-S) test, and the results indicated that none of the continuous variables followed a normal distribution. Therefore, rank-sum tests were used for comparison, as shown in Table 1.

### 3.3 Binary logistic regression analysis of influencing factors of ENFI in ICU patients

Based on the results of the univariate analysis, 21 risk factors with *p* < 0.05 were selected as independent variables, including seven continuous variables and 14 categorical variables. Among the seven continuous variables, one was related to general information, and six were related to observational data. Among the 14 categorical variables, one was related to general information, and the rest were related to observational data. These variables were included in the logistic regression analysis, and the results are detailed in Tables 2, 3.

**Table 3:** Logistic regression analysis of factors influencing ENFI in ICU patients (*N* = 487).

Risk factor	Regression coefficient	Standard error	OR	95%CI		*P*
				Lower limit	Upper limit	
APACHEIIscore	0.239	0.05	1.27	1.152	1.4	<0.001
IAP (mmHg)	1.338	0.198	3.811	2.586	5.617	<0.001
Blood glucose (mmol/L)	0.247	0.073	1.28	1.109	1.478	0.001
Mechanical ventilation	−3.172	0.774	0.042	0.009	0.191	<0.001
Early enema	1.516	0.612	4.554	1.372	15.113	0.013
Use of analgesics	2.051	0.613	7.772	2.339	25.831	0.001
Use of probiotics	−1.499	0.639	0.223	0.064	0.781	0.019

### 3.4 Model construction and evaluation

#### 3.4.1 Model construction

We used Python 3.9 to build and evaluate the prediction models based on the results of the influencing factor analysis. The first step in the machine learning process was to import the model-selection module and initialize the environment by defining the data frame, target variable (feeding tolerance/intolerance), training set (80%), and test set (20%). Bootstrap stability verification was performed on the included features. The stability of the selected frequency for each feature in the resampling is greater than 95%, indicating that the importance of the selected feature is highly confident. To ensure the generalization ability of the model and avoid data leakage, the hierarchical random segmentation strategy was used to divide the dataset to ensure that all preprocessing (e.g., imputation, normalization) was only fitted on the training set. We used grid search and cross-validation on the training set to enhance the model’s performance and lower the likelihood of overfitting. We divided the dataset into an 80% training set and a 20% test set. Models were built using three algorithms: LR, SVM, and RF.

#### 3.4.2 Evaluation of training set models

**Figure 3:**
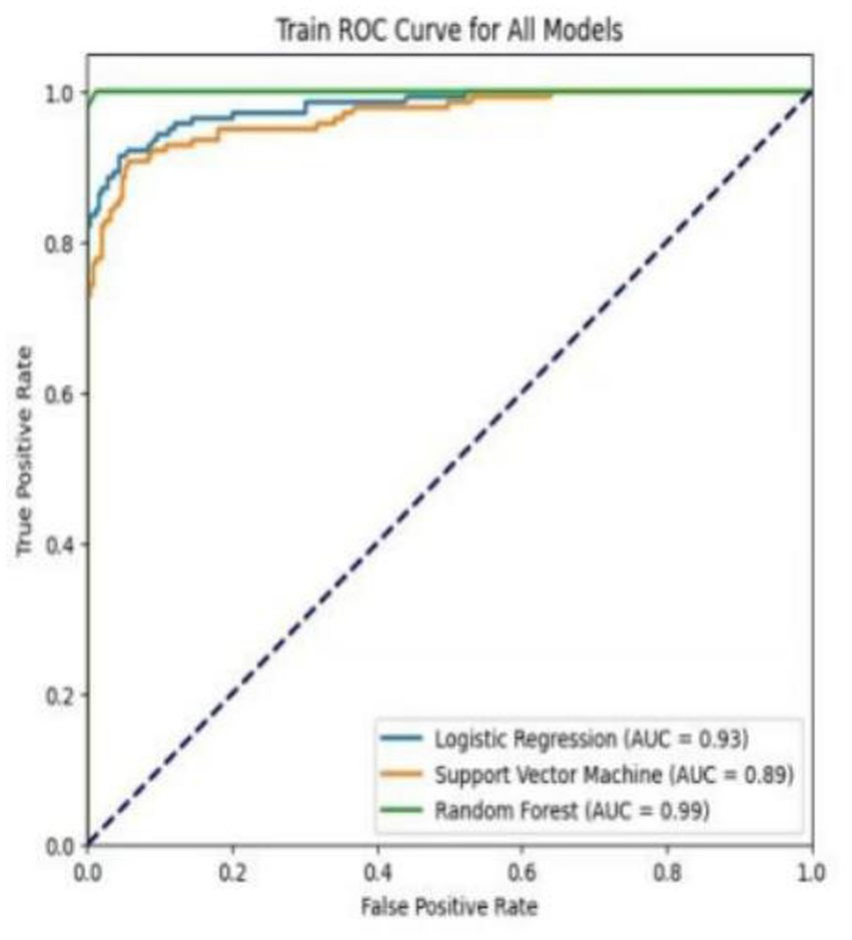
ROC curves of three models in the training set.

**Figure 4:**
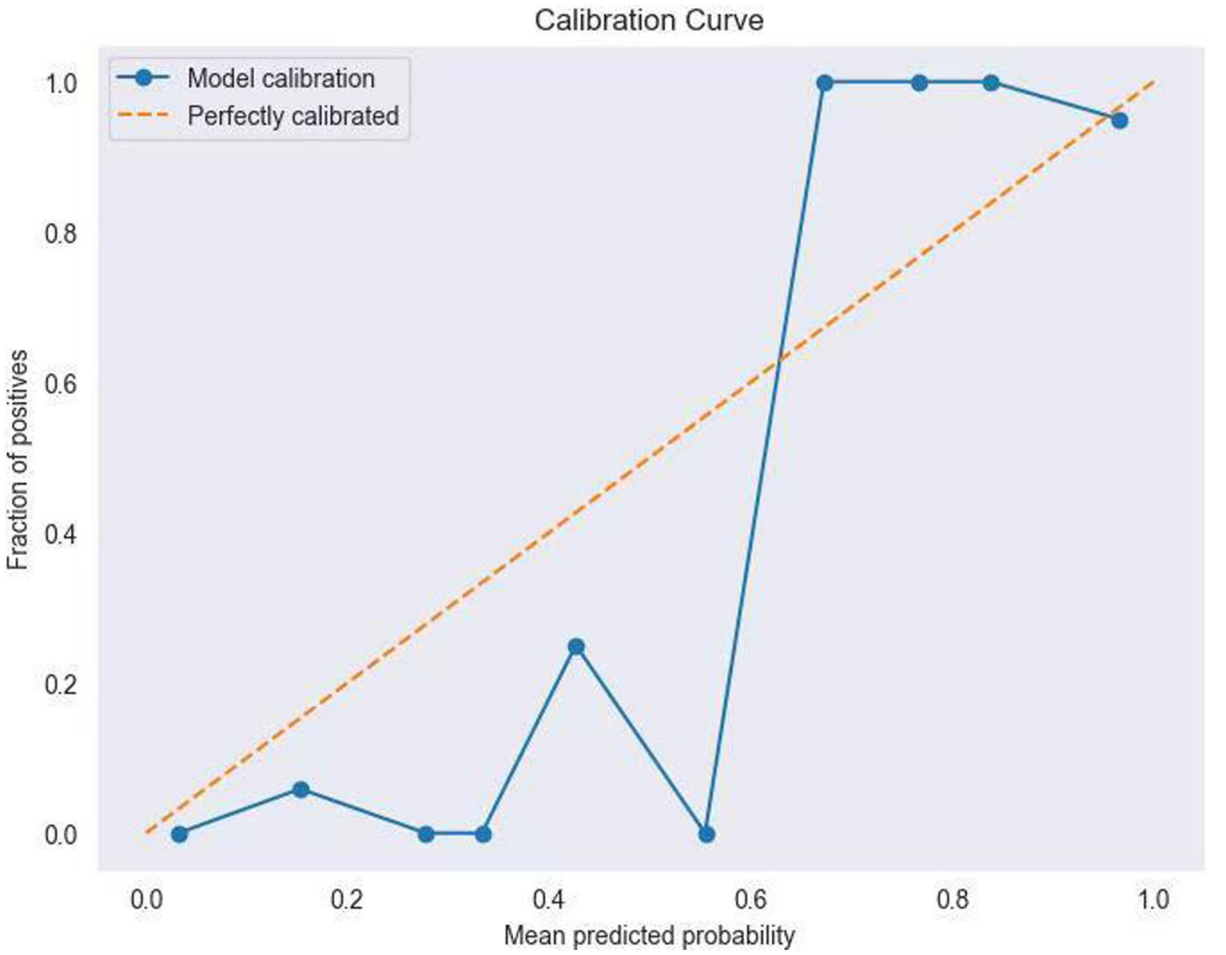
Calibration curve of the Random Forest model.

Table 4 shows the performance metrics on the training set for the three models built using different methods. Among them, the AUC of the RF model is 0.9511, and the F1 score is 0.9446, achieving the highest scores among the three groups of models. The ROC curves for each model on the training set are shown in Figure 3. We evaluated the model’s calibration using the calibration curve Brier score. Figure 4 displays the results of the calibration curve, which visualizes the model’s calibrability. The Brier score (0.0463) is a direct measure of probabilistic prediction accuracy, indicating that the model is well calibrated overall.

**Table 4:** Performance metrics of the three methods of model building for the training set.

Model	AUC	Accuracy	Precision	Recall	F1 score
RF	0.951	0.961	0.977	0.914	0.945
SVM	0.924	0.941	0.968	0.846	0.913
LR	0.931	0.943	0.954	0.886	0.919

#### 3.4.3 Model validation and evaluation

**Figure 5:**
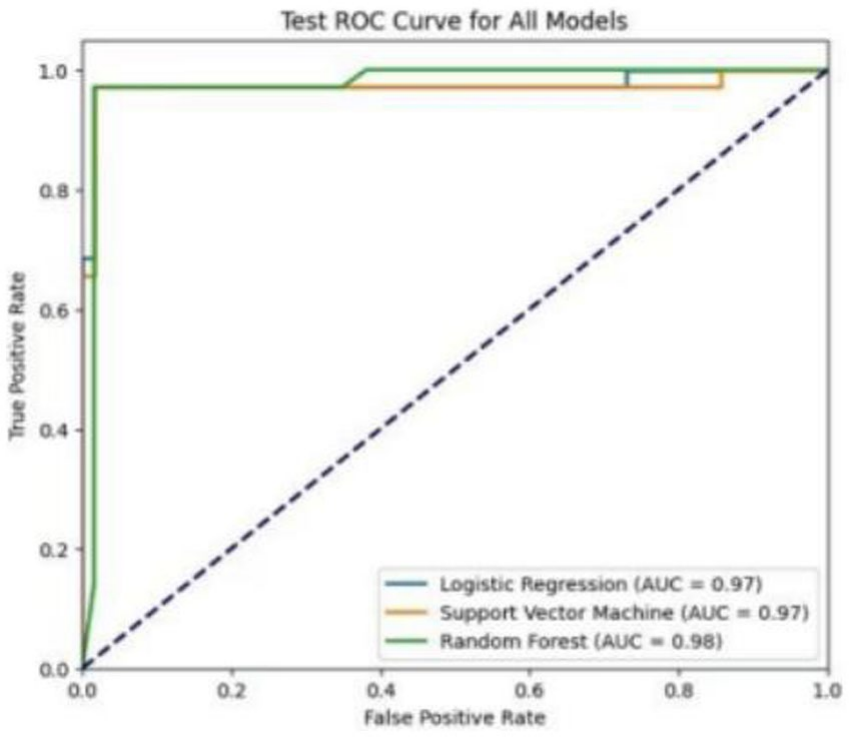
ROC curves comparing model performance in the test set.

We used resampling methods to validate the three models. The results of the test set showed that the RF model AUC of 0.982. Comprehensive analysis indicated that the RF model performed the best on the test set, as detailed in Table 5. The ROC curves for each model in the test set are shown in Figure 5.

**Table 5:** Comparison of test set model performance (*N* = 98).

Model	AUC	Accuracy	Precision	Recall	F1 score
RF	0.982	0.959	0.943	0.943	0.943
SVM	0.975	0.980	0.971	0.971	0.971
LR	0.979	0.929	0.868	0.943	0.904

### 3.5 Feature importance ranking of ENFI influencing factors in ICU patients

**Figure 6:**
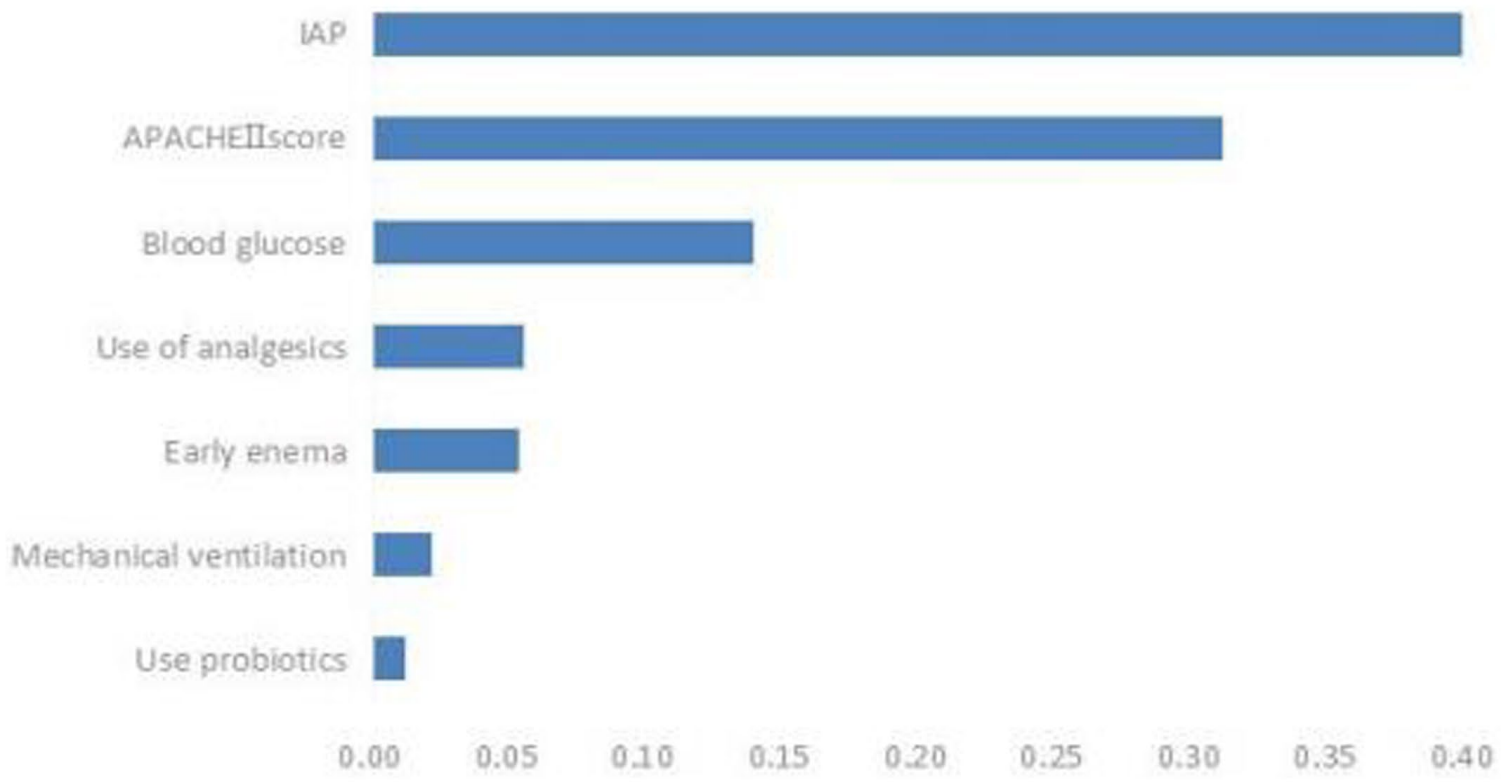
Key predictors of ENFI in ICU patients in order of importance.

Based on the comparison of the models, the RF model demonstrated the best overall performance and provided a ranking of feature importance. The seven influencing factors for ENFI occurrence, ranked in descending order of importance, include intra-abdominal pressure, APACHE II score, blood glucose level, and use of analgesics, among others, as illustrated in Figure 6.

## 4 Discussion

### 4.1 Incidence of ENFI

This study included 487 ICU patients, of whom 175 experienced ENFI, accounting for 35.9% of the total. This figure is close to the 38% incidence rate reported by other scholars (16). This high rate highlights the urgent need for proactive identification and management of ENFI to mitigate complications such as prolonged ICU stays and malnutrition.

### 4.2 Influencing factors of ENFI

There are numerous factors influencing ENFI in ICU patients, and continuous exploration is needed. This study identified the following conclusions: The APACHE II score, intra-abdominal pressure, blood glucose level, mechanical ventilation, and several other factors independently influence early ENFI in ICU patients.

#### 4.2.1 APACHE II score

The APACHE II score is an authoritative indicator for assessing the severity of illness and predicting prognosis in ICU patients. It is widely used in clinical practice, with higher scores indicating more severe illness and a worse prognosis (17). The more severe the patient’s condition, the more intense the systemic stress response, leading to pronounced vasoconstriction of splanchnic vessels, gastrointestinal mucosal ischemia, and even erosion, thereby impairing gastrointestinal function. On the other hand, the body enters a hypercatabolic state, triggering the breakdown, consumption, and loss of tissue proteins, resulting in hypoalbuminemia. This condition induces gastrointestinal mucosal edema, further exacerbating mucosal injury and ultimately reducing enteral nutrition tolerance in ICU patients (18). It was found that the intolerance group’s APACHE II score (33.29 ± 10.41) was significantly higher than the tolerance group’s (18.98 ± 6.41). Statistical analysis also confirmed that the APACHE II score is a separate risk factor for ENFI in ICU patients (*p* < 0.05), which is in line with what other research has found (2). Routine APACHE II scoring within 24 h of ICU admission can stratify high-risk patients, prompting closer EN monitoring and early interventions.

#### 4.2.2 Intra-abdominal pressure

In this study, the IAP in the tolerance group was 7.29 ± 1.70 mmHg, while in the intolerance group, it was 11.02 ± 1.96 mmHg. In the intolerance group, 82 patients (46.86%) had intra-abdominal hypertension (IAP > 12 mmHg), but no cases of abdominal compartment syndrome (IAP > 20 mmHg) were observed. The fact that most patients suffered from respiratory or neurological diseases may explain this phenomenon. Compared to patients with gastrointestinal diseases, the increase in IAP in these patients was relatively mild, but the IAP in the intolerance group was still significantly higher than that in the tolerance group.

IAP (19) refers to the pressure within the abdominal cavity, and its increase can be attributed to factors such as increased organ volume, increased fluid volume, and the use of mechanical ventilation. The gastrointestinal tract is one of the most sensitive organs to increased IAP. As IAP rises, mesenteric blood flow decreases, and venous return is obstructed, leading to intestinal edema and impaired intestinal function, resulting in gastrointestinal adverse effects (20). Clinically, we often estimate IAP by measuring gastric, superior vena cava, inferior vena cava, or bladder pressure. Bladder pressure is considered the “gold standard” for IAP monitoring due to its simplicity, non-invasiveness, accuracy, and minimal influence by human factors or the disease itself (21). This study also used bladder pressure as a proxy for IAP. Healthcare providers should regularly monitor IAP in clinical practice and actively seek causes and interventions for patients with high IAP.

#### 4.2.3 Blood glucose level

In this study, the blood glucose level in the intolerance group was 9.34 ± 3.50 mmol/L, higher than that in the tolerance group (7.34 ± 3.33 mmol/L). For critically ill patients, hyperglycemia may result not only from pre-existing diabetes but also from various other factors (22). Stress-induced hyperglycemia refers to elevated blood glucose levels in patients without a history of diabetes, occurring in response to severe trauma, shock, cardiovascular accidents, or other stressors (23). Elevated blood glucose levels can reflexively reduce the tension of the gastric antrum smooth muscle, leading to decreased gastric motility and symptoms such as gastric retention. Furthermore, high blood glucose can make the pylorus work harder, which can make the contractions of the stomach and duodenum not work together properly. Such conditions can cause problems with emptying the stomach and greatly raise the risk of ENFI (18, 28). Therefore, healthcare providers should pay close attention to blood glucose monitoring in critically ill ICU patients. When hyperglycemia occurs, appropriate measures should be taken to maintain blood glucose within a relatively stable range, which can help reduce the incidence of ENFI.

#### 4.2.4 Mechanical ventilation

The results of this study indicate that ICU patients on mechanical ventilation are more likely to experience ENFI, and the use of mechanical ventilation is a risk factor for ENFI in ICU patients (*p* < 0.05). Mechanical ventilation is an artificial support system that controls or alters a patient’s spontaneous breathing movements. Its purpose is to maintain airway patency, improve ventilation and oxygenation, and prevent carbon dioxide retention and hypoxia. It is a common treatment method for critically ill patients in clinical practice (24). High levels of positive end-expiratory pressure (PEEP) can cause organs around the heart to have poor blood flow, lower cardiac output, and gastrointestinal ischemia. Such condition can slow the movement of food through the digestive tract or damage the mucosa, which can set off ENFI. On the other hand, mechanical ventilation can cause gas to enter the stomach or lead to bile reflux, further increasing IAP and affecting the patient’s tolerance to enteral nutrition. Therefore, in real life, IAP monitoring should be done regularly on patients on mechanical ventilation before and after they start enteral nutrition so that targeted measures can be taken in time if ENFI happens. Additionally, energy expenditure can be estimated based on carbon dioxide production calculated by the ventilator, and individualized feeding plans can be developed based on the patient’s energy expenditure to reduce the occurrence of ENFI (25).

### 4.3 Model evaluation

Research on enteral nutrition feeding intolerance (ENFI) prediction has been conducted for many years (27). This study developed prediction models for risk factor screening by using conventional clinical data, which were analyzed through univariate analysis and logistic regression, while employing three machine learning algorithms. The three machine learning algorithms—LR, SVM, and RF—each exhibit distinct advantages and limitations in predicting enteral nutrition feeding intolerance (ENFI) in ICU patients. LR offers the best interpretability, providing clinically actionable odds ratios, but its linearity assumption may overlook complex interactions among risk factors. SVM captures nonlinear relationships through kernel functions, yet its “black-box” nature and sensitivity to class imbalance limit its clinical utility. Our Random Forest (RF) model achieves superior performance (AUC = 0.9511) by processing high-dimensional nutrient-specific data and automatically detecting feature interactions—albeit with the need for careful hyperparameter tuning due to its ensemble structure—findings that are consistent with and extend the benefits of ML as demonstrated by Ong et al. (26) in the context of ventilator management, collectively underscoring the transformative potential of machine learning in different predictive domains in the ICU (26).

To enhance the clinical interpretability of RF, this study employs a feature importance ranking method, intuitively illustrating the contribution of each feature to individual patient predictions, thereby facilitating clinical comprehension. The analysis confirms intra-abdominal pressure as the most critical predictor, aligning with established physiological mechanisms of ENFI pathogenesis. By bridging the gap between algorithmic predictions and clinical decision-making, this understandable AI framework enables clinicians to access not only risk scores but also their underlying determinants, fostering trust and promoting implementation in critical care settings. This approach adheres to current standards for transparent AI in healthcare, demonstrating a reproducible method for deploying machine learning tools in clinical environments where interpretability is paramount.

Simultaneously, we fully acknowledge concerns about potential overfitting due to the high AUC values in clinical prediction models. To this end, we systematically optimize and validate the model development process, and the results show that we use grid search + 5-fold cross-validation to tune the model parameters, and through parameter tuning and rigorous validation, the AUC of the training set decreases by 0.041, and the AUC of the test set maintains at 0.981, with the difference between the two values of 0.031, which is much lower than the threshold for hinting at overfitting in the clinical prediction model, and the model maintains high performance while overfitting.

These results indicate that the optimized model achieves high performance while minimizing the risk of overfitting. The stable performance on the test set further demonstrates that the model captures true predictive signals rather than noise.

In terms of clinical applicability, the Brier score (0.0463) and calibration curve of the optimized model demonstrated good overall calibration, especially in the high-risk interval (predicted probability > 0.8) where it was in high agreement with the ideal curve. This finding holds significant clinical relevance: when the model predicts an ENFI probability≥ 80%, clinicians can confidently initiate parenteral nutrition support to avoid complications from feeding intolerance. However, in the intermediate-risk range (0.4–0.6), deviations between predicted probabilities and observed frequencies suggest the need for integrating additional clinical indicators (e.g., gastric residual volume monitoring, bowel sound assessment) for comprehensive decision-making.

While current static models have shown strong predictive performance, we recognize that these models may not fully capture the time-series dynamics inherent in the condition of ICU patients. To enhance the timeliness and clinical relevance of the models, future work will focus on developing a dynamic prediction framework that incorporates longitudinal parameters. This approach will take full advantage of the complementary strengths of the Long Short-Term Memory (LSTM) network for analyzing temporal patterns and the Random Forest (RF) algorithm for dealing with static features, while integrating real-time data collection through the Hospital Information System (HIS) to create a comprehensive closed-loop “monitor-predict-intervene” management system.

## 5 Limitations and future directions

Although the risk of overfitting was reduced by parameter tuning and calibration analysis, this study was still a single-center retrospective analysis, and the generalization of the model for cross-institutional and cross-population data needs to be further verified. The current model was developed using retrospective data from an ICU in a tertiary hospital in East China (*N* = 487), and its clinical application may be subject to several constraints. First, the training data predominantly originated from high-level medical centers in a specific region, potentially limiting its generalizability to diverse healthcare settings with varying institutional levels, heterogeneous population characteristics, and distinct clinical protocols. Second, while internal validation demonstrated satisfactory performance, the model’s robustness in real-world scenarios necessitates rigorous external validation. Therefore, we suggest using this model as a secondary decision-support tool in clinical practice, complementing physicians’ clinical judgment.

To address these limitations, we propose a multicenter prospective validation study to systematically evaluate the model’s external validity. This investigation will enroll ICU patients from 12 hospitals of different tiers across five geographical regions (East, North, South, West, and Central China; projected sample size *N* = 1,500), implementing standardized prospective data collection protocols. The study will specifically examine: (1) predictive stability across varying healthcare resource allocations; (2) applicability in ethnically and geographically diverse populations; and (3) robustness in heterogeneous clinical practice environments. Scheduled for initiation in the second quarter of 2026 with an 18-month duration, the study will employ standardized inter-center quality control measures and regular data coordination meetings to ensure data comparability and reliability.

After completing the external validation, the integration of the predictive model with the hospital electronic health record (EHR) system has a promising application, but still faces many challenges at the technical and clinical levels. In terms of technical implementation, it is necessary to develop standardized API interfaces to interface with mainstream EHR platforms (e.g., Epic) to achieve automatic extraction of key parameters such as APACHE II scores, and at the same time, adopt a containerized deployment scheme to take into account computational efficiency, compatibility of data architectures, and privacy compliance requirements such as the Protection of Individual Personal Information Law (PIPL). In the future, by integrating the time series analysis capability of LSTM network and the feature processing advantage of Random Forest (RF) algorithm, and combining with the real-time data collection function of Hospital Information System (HIS), we can build a more complete dynamic prediction framework, and eventually form a closed-loop management system of “Monitoring-Prediction-Intervention,” which will significantly improve the timeliness and clinical relevance of the model. In terms of clinical implementation, the system will provide visual risk warning (e.g., traffic light indicators) and intelligent decision support (e.g., automated feeding regimen suggestions), and ensure the successful application of the prediction model in real-life healthcare scenarios through the seamless integration with existing clinical workflows, as well as the layered training program, system optimization feedback mechanism, and multidisciplinary collaboration mechanism.

## Data Availability

The raw data supporting the conclusions of this article will be made available by the authors, without undue reservation.
